# Supplementary material for: A Cross-Sectional Survey of Musculoskeletal Injuries in South African Shotokan Karate
Source: J Funct Morphol Kinesiol. 2025 Nov 27;10(4):463. doi: 10.3390/jfmk10040463 (PMC12734262; doi:10.3390/jfmk10040463)
Supplement: Supplementary file 1 [file jfmk-10-00463-s001.zip › jfmk-3937492-supplementary.pdf]

**Section A: Participant Demographics**

1. Age in years

| |
|---|
| |

2. Weight in kilograms

| |
|---|
| |

3. Height in centimetres

| |
|---|
| |

4. Gender

| | |
|---|---|
| Male | 1 |
| Female | 2 |
| Prefer not to say | 3 |

**Section B: General activity level/ IPAQ (Short Form)**

5. During the last 7 days, did you do **vigorous** physical activities like heavy lifting, digging, aerobics, or fast bicycling?

*Vigorous physical activities refer to activities that take hard physical effort and make you breathe much harder than normal.

| | |
|---|---|
| Yes | 1 |
| No | 2 |

6. If yes to question 5: During the last 7 days, on how many days did you do **vigorous** physical activities like heavy lifting, digging, aerobics, or fast bicycling?

______days per week

7. If yes to question 5: How much time did you usually spend doing **vigorous** physical activities on one of those days?

For example, if you spend 2.5 hours per day doing vigorous physical activities, your answer will be 2 hours and 30 minutes per day.

____ hours per day

_____ minutes per day

8. During the last 7 days, did you do **moderate** physical activities like carrying light loads, bicycling at a regular pace, or doubles tennis? Do not include walking.

*Moderate activities refer to activities that take moderate physical effort and make you breathe somewhat harder than normal.

| | |
|---|---|
| Yes | 1 |
| No | 2 |

9. If yes to question 8: During the last 7 days, on how many days did you do **moderate** physical activities?

______days per week

10. If yes to question 8: How much time did you usually spend doing **moderate** physical activities on one of those days?

For example, if you spend 2.5 hours per day doing moderate physical activities, your answer will be 2 hours and 30 minutes per day.

____ hours per day

_____ minutes per day

11. During the last 7 days, did you do any **walking**?

| | |
|---|---|
| Yes | 1 |
| No | 2 |

12. If yes to question 11: During the last 7 days, on how many days did you **walk** for at least 10 minutes at a time?

____ days per week

13. If yes to question 11: How much time did you usually spend **walking** on one of those days?

For example, if you spend 2.5 hours per day walking, your answer will be 2 hours and 30 minutes per day.

____ hours per day

_____ minutes per day

14. During the last 7 days, do you know how much time did you spend **sitting** on a week day? Include time spent at work, at home, while doing course work and during leisure time. This may include time spent sitting at a desk, visiting friends, reading, or sitting or lying down to watch television.

| | |
|---|---|
| Yes | 1 |
| No | 2 |
| Don't know/ not sure | 3 |

1. If yes to question 14: During the last 7 days, how much time did you spend **sitting** on a week day? Include time spent at work, at home, while doing course work and during leisure time. This may include time spent sitting at a desk, visiting friends, reading, or sitting or lying down to watch television.

For example, if you spend 2.5 hours per day sitting, your answer will be 2 hours and 30 minutes per day.

____ hours per day

____minutes per day

**Section C: Karate background**

2. How many years have you trained karate?

| | |
|---|---|
| Less than 1 year | 1 |
| 1-2 years | 2 |
| 3-5 years | 3 |
| 6-10 years | 4 |
| 11-15 years | 5 |
| 16-20 years | 6 |
| More than 20 years | 7 |

3. How many days a week do you train karate?

| | |
|---|---|
| 1 day per week | 1 |
| 2 days per week | 2 |
| 3 days per week | 3 |
| 4 days per week | 4 |
| 5 days per week | 5 |
| 7 days per week | 6 |

4. How long is a karate training session normally?

| | |
|---|---|
| Less than 30 minutes | 1 |
| 30-60 minutes | 2 |
| 1 - 1.5 hours | 3 |
| 1.5 - 2 hours | 4 |
| More than 2 hours | 5 |

**Section D: Karate Injuries**
**These may include any injuries sustained to muscles, bones, ligaments, tendons or muscles either on impact or overtime.**

5. Have you ever sustained an injury from doing karate?

| | |
|---|---|
| Yes | 1 |
| No | 2 |

The following questions will be answered if you sustained an injury from doing karate. If you did not sustain an injury, thank you for answering the questionnaire.

6. How many injuries have you sustained from doing karate?

| | | |
|---|---|---|
| 21.1 | 1 injury | 1 |
| 21.2 | 2 injuries | 2 |
| 21.3 | 3 injuries | 3 |
| 21.4 | 4+ injuries | 4 |

7. What was your belt ranking in Shotokan karate when you sustained an injury? If more than one injury occurred, please mark all applicable.

| | |
|---|---|
| White belt | 1 |
| Yellow belt | 2 |
| Orange belt | 3 |
| Green belt | 4 |
| Blue belt | 5 |
| Purple belt | 6 |

| | |
|---|---|
| Red belt | 7 |
| Brown belt 1 | 8 |
| Brown belt 2 | 9 |
| Brown belt 3 | 10 |
| 1st Dan Black belt (Shodan) | 11 |
| 2nd Dan Black belt (Nidan) | 12 |
| 3rd Dan Black belt (Sandan) | 13 |
| 4th Dan Black belt (Yondan) | 14 |
| 5th Dan Black belt (Godan) | 15 |
| 6th Dan Black belt (Rokudan) | 16 |
| 7th Dan Black belt (Shichidan) | 17 |
| 8th Dan Black belt (Hachidan) | 18 |
| 9th Dan Black belt (Kudan) | 19 |
| 10th Dan Black belt (Judan) | 20 |

8. Was the injury/ies sustained during a karate tournament or training? If you've had more than one injury, mark the applicable option for all your injuries.

| | | |
|---|---|---|
| 22.1 | Tournament/competition | 1 |
| 22.2 | Training | 2 |
| 22.3 | Both | 3 |

9. Was your injury/ies sustained on impact or did it develop over time? If you've had more than one injury, mark the applicable option for all your injuries.

| | | |
|---|---|---|
| 23.1 | Immediately | 1 |
| 23.2 | Over-time | 2 |
| 23.3 | Both | 3 |

10. If you had any injuries that occurred immediately, at the time of the injury/ies, which protective equipment were you wearing? Mark all applicable

| | | |
|---|---|---|
| 24.1 | Gloves | 1 |
| 24.2 | Gi | 2 |
| 24.3 | Shin protectors | 3 |
| 24.4 | Foot protectors | 4 |
| 24.5 | Chest protector | 5 |
| 24.6 | Breast protector | 6 |
| 24.7 | Gum guard | 7 |
| 24.8 | Face mask | 8 |
| 24.9 | Groin guard | 9 |
| 24.10 | Did not wear any equipment | 11 |

11. Which area(s) of the body was/were injured in karate? If you've had more than one injury, mark all applicable options.

| | Region | Tick if area was injured |
|---|---|---|
| 25.1 | Head/face | 1 |

| | | |
|---|---|---|
| 25.2 | Jaw | 2 |
| 25.3 | Neck | 3 |
| 25.4 | Chest | 4 |
| 25.5 | Abdomen | 5 |
| 25.6 | Mid-back | 6 |
| 25.7 | Low-back | 7 |
| 25.8 | Shoulder | 8 |
| 25.9 | Upper arm | 9 |
| 25.10 | Elbow | 10 |
| 25.11 | Forearm | 11 |
| 25.12 | Wrist | 12 |
| 25.13 | Hand | 13 |
| 25.14 | Hip | 14 |
| 25.15 | Buttock | 15 |
| 25.16 | Thigh | 16 |
| 25.17 | Groin | 17 |
| 25.18 | Knee | 18 |
| 25.19 | Lower leg | 19 |
| 25.20 | Ankle | 20 |
| 25.21 | Foot | 21 |
| 25.22 | Other (specify) | 22 |

12. Which type of injury/ies did you sustain while doing karate?

| | | |
|---|---|---|
| 26.1 | Bone fracture | 1 |
| 26.2 | Dislocation | 2 |
| 26.3 | Ligament Injury (sprain) | 3 |
| 26.4 | Muscle injury (strain) | 4 |
| 26.5 | Tendon injury | 5 |
| 26.6 | Bruise | 6 |
| 26.7 | Laceration (cut) | 7 |
| 26.8 | Loss of consciousness | 8 |
| 26.9 | Not sure | 9 |
| 26.10 | Other (specify) | 10 |

13. What was the **longest** time that an injury prevented you from training?

| | |
|---|---|
| I trained with all my injuries | 1 |
| A few days | 2 |
| 1-2 weeks | 3 |
| 3 weeks | 4 |
| 1 month | 5 |
| 2-3 months | 6 |
| 4-6 months | 7 |
| 6 months-1 year | 8 |
| Longer than 1 year | 9 |

14. Was your injury/ies treated by any of the following healthcare professionals? If you've had more than one injury, mark all applicable options.

| | | |
|---|---|---|
| 28.1 | I was treated by a General practitioner (GP) | 1 |
| 28.2 | I was treated by a Chiropractor | 2 |
| 28.3 | I was treated by a Physiotherapist | 3 |
| 28.4 | I was treated by a Biokineticist | 4 |
| 28.5 | I was treated by an Orthopaedic surgeon | 5 |
| 28.6 | I treated myself (rest, ice, medication etc.) | 6 |
| 28.7 | I didn't receive any treatment | 7 |
| 28.8 | Other (specify) | 8 |

15. What was the extent of treatment for your **most severe** injury?

| | |
|---|---|
| I required no treatment | 1 |
| I treated myself | 2 |
| I was diagnosed and sent home to recover | 3 |
| I required some treatment and rehabilitation by a healthcare professional | 4 |
| I was hospitalised for my injury | 5 |
| I required surgery for my injury | 6 |
| Other (specify) | 7 |

16. Has anyone recommended Chiropractic treatment to you during your time of practising karate?

| | |
|---|---|
| Yes | 1 |
| No | 2 |

17. If yes, who recommended Chiropractic treatment to you?

| | |
|---|---|
| Family/friend | 1 |
| Karate instructor (sensei) | 2 |
| Other medical professionals | 3 |
| Other (specify) | 4 |

*** End of questionnaire, thank you for your time***
